# LncRNA DANCR contributes to tumor progression via targetting miR-216a-5p in breast cancer: lncRNA DANCR contributes to tumor progression

**DOI:** 10.1042/BSR20181618

**Published:** 2019-04-23

**Authors:** Weiyang Tao, Chunyang Wang, Bifa Zhu, Guoqiang Zhang, Da Pang

**Affiliations:** 1 Department of Breast Surgery, The Third Affiliated Hospital of Harbin Medical University, Harbin City, Heilongjiang Province, P.R. China; 2 Department of Urology, The First Affiliated Hospital of Harbin Medical University, Harbin City, Heilongjiang Province, P.R. China

**Keywords:** LncRNA DANCR, miRNA-216a-5p, triple negative breast cancer, tumor progression

## Abstract

Breast cancer, the most frequently occurring malignant tumor, has high mortality rate, especially triple-negative breast cancer (TNBC). LncRNA-differentiation antagonizing non-protein coding RNA (lncRNA DANCR) has been found that its aberrant expression was associated with tumor progression and it was promising to be a potential target for cancer therapy. The goal of the present study was to explore the biological effects and underlying mechanism of DANCR in breast cancer. Our results showed that DANCR was up-regulated in TNBC tissues and breast cancer cells compared with normal breast tissues and cells, and higher DANCR level suggested poorer prognosis, implying that it was promising to be a novel biomarker used for TNBC diagnosis and prognosis. To better research the functions and mechanism of DANCR on breast cancer cells, we selected two cell lines used for next study: one TNBC cell line–MDA-MB-231 and one ER-positive breast cancer cell line–MCF-7. Further study indicated that DANCR overexpression significantly promoted cell proliferation and invasion *in vitro* and contributed to tumor growth *in vivo*. To deeply understand its molecular mechanism, miRNA-216a-5p was identified as a target of DANCR by bioinformatic analysis. Experiments demonstrated that miRNA-216a-5p interacted with DANCR and its inhibitor could weaken the influences induced by DANCR knockdown for cancer cells, including cell proliferation and invasion, and the expression of Nanog, SOX2, and OCT4. Therefore, DANCR might act as a tumor promoter by targetting miRNA-216a-5p, which might provide a potential therapy target for breast cancer treatment.

## Introduction

Globally, breast cancer is the most frequently occurring malignant tumor for females, with about 1.7 million new diagnosed patients and 522,000 breast cancer-caused deaths every year [1–3]. Based on the molecular expression pattern of estrogen receptor (ER), progesterone receptor (PR), human epidermal growth factor receptor 2 (HER-2), and Ki67, breast cancer can be divided into five subtypes [4]. Triple-negative breast cancer (TNBC) is one of the most aggressive subtypes of breast cancer in clinical and defined as lack of ER, PR, and HER-2, which accounts for nearly 15% of all breast cancer patients [5–7]. At present, great advancements have been made in molecular diagnosis and targetted therapy, which contribute to the obvious increase of survival rate of patients with breast cancer. However, some targetted treatments have bad therapeutic effects for the patients with TNBC, such as endocrine therapy and HER2-targetted trastuzumab, because there are no appropriate targets of these drugs in their body [8]. In addition, conventional chemotherapy, as a standard treatment used for TNBC patients, often lead to the development of chemotherapy resistance and then cause recurrence and death for patients [9]. Thus, patients with TNBC have relative worse prognosis than those with other breast cancer subtypes. Moreover, the understanding of molecular mechanisms is still poor for TNBC, which makes it very hard to propose new treatment strategies. Therefore, it is very essential to find new molecular biomarkers related with TNBC progress or prognosis in order to provide better personalized treatments and improve clinical outcome for TNBC patients.

LncRNAs, one subtype of ncRNAs, are characterized with length more than 200 nts and have limited or no protein coding ability [10]. Plenty of experiments have proved that lncRNAs participated various cellular processes by regulating gene expression, such as cell cycle regulation, cell proliferation and differentiation, nuclear-cytoplasmic trafficking, and gene transcription and translation [11–14]. Furthermore, many evidences displayed that lncRNAs were abnormally expressed in variety of diseases, implying that they probably play key roles on many diseases especially cancers [10]. For example, lncRNA HOXA11-AS was overexpressed in gastric cancer and played promoting functions on cell proliferation and invasion by scaffolding several chromatin modification factors [15]; for bladder cancer, lncRNA-LOC572558 could inhibit cell proliferation and tumor growth through regulating AKT-MDM2-p53 signaling pathway [16]; and high level of lncRNA FAL1 acted as a CeRNA of miR-1236 to promote proliferation and migration of hepatocellular carcinoma cells [17]. Despite many lncRNAs have been found and studied, the knowledge of their functions on TNBC is still lacking.

LncRNA-differentiation antagonizing non-protein coding RNA (DANCR), a novel identified lncRNA from human cancers, is located on chromosome 4. Recently, some studies have reported that lncRNA DANCR is up-regulated in various human cancers and its aberrant expression could promote tumor progression by improving cancer cell proliferation and invasion, such as glioma, non-small cell lung cancer, and prostate cancer [18–20]. Based on previous reports, we speculated that DANCR had potential oncogenic peculiarity. Nevertheless, the DANCR biological effects and underlying mechanism is still unclear in TNBC. In our work, we selected two breast cancer cell lines: one TNBC cell line–MDA-MB-231 and one ER-positive breast cancer cell line–MCF-7, in order to study DANCR functions on breast cancer and explore whether it was specific for TNBC. First, DANCR expression level was examined in breast cancer tissues and paired adjacent non-tumor tissues both obtained from 57 TNBC patients. The relationship between DANCR level and survival rate of patients was analyzed. Then cell transfection assay was performed to stably change DANCR expression in two breast cancer cells (MCF-7, MDA-MB-231), following CCK-8, and transwell assay were used to explore its functions on breast cancer cell proliferation and invasion *in vitro*. Xenograft animal study was used to further confirm DANCR effects on tumor progression. Moreover, we explored its possible molecular mechanism in breast cancer.

## Materials and methods

### Breast cancer tissue collection

The present study was approved by the Ethics Committees of The Third Affiliated Hospital of Harbin Medical University, and all patients have signed consent form. The research has been carried out in accordance with the World Medical Association Declaration of Helsinki. A total of 57 pairs of breast cancer tissues and paired adjacent non-tumor tissue were collected from patients with TNBC in The Third Affiliated Hospital of Harbin Medical University from August 2012 to December 2014. Tissue samples were immediately put into liquid nitrogen and stored at −80°C.

### Cell culture

Human breast cancer cell lines (MCF-7, MDA-MB-231) and normal human breast epithelial cell (MCF-10A) were purchased from ATCC (Manassas, VA, U.S.A.). All cells were cultured in RPMI-1640 medium (Hyclone, U.S.A.) supplemented with 10% fetal bovine serum (Hyclone, U.S.A.) in an incubator at 37°C with 5% CO_2_.

### BLAST alignment

It was well known that RNA transcripts can have multiple exons aligning to different non-contiguous regions of a chromosome. Alignment searches were performed with NCBI’s BLAST suite. For alignment to RNA sequences, the top search result with a value <0.01 was reported. By analysis, we chose miRNA-216a-5p (miR-216a) used for further experiments because it was highly combined with DANCR.

### RNA extraction and qRT-PCR

Total RNA was extracted from tissues and cells using a miRNeasy Mini Kit (Qiagen, Valencia, CA, U.S.A.) in accordance with the manufacturer’s instructions, immediately the concentration and quality of RNA were detected by NanoDrop 2000 (Thermo Fisher, Wilmington, DE, U.S.A.). Then, the obtained RNA was used to synthesize the first-strand complementary DNA (cDNA), using TransScript first-strand cDNA synthesis SuperMix (TransGen, Beijing, China) as per the manufaturer’s protocol. RT-PCR experiment was performed with SYBR green qPCR SuperMix (Applied Biosystems Life Technologies, Foster, CA, U.S.A.) in ABI prism 7500 sequence detection system (Applied Biosystems Life Technologies, Foster, CA, U.S.A.). Experimental conditions were as below: 55°C for 10 min, 40 cycles of 95°C for 30 s, 55–59°C for 30 s, and 72°C for 42 s. Cycle threshold (Ct) values were detected and used to calculate the relative expression levels of DANCR and miR-216a according to 2^−ΔΔ*C*^_T_ method. GADPH and U6 were respectively as loading control of DANCR and miR-216a.

### Oligonucleotide transfection

Oligonucleotides sequences were chemically synthesized by GenePharma (Shanghai, China), including siRNA against DANCR (si-DANCR), miR-216a inhibitor (216a-inhibitor), miR-216a mimics (216a-mimics), and their negative controls (NCs). Oligonucleotide transfection was performed using Lipofectamine 2000 (Invitrogen, Carlsbad, CA, U.S.A.) according to the manufacturer’s protocol. The cells without transfection were as control group. After transfection, the expression level of DANCR or miR-216a was measured by qRT-PCR.

### Luciferase reporter assay

First, oligonucleotide sequences containing DANCR cDNA fragment (including miRNA binding sites) were amplified and cloned into the pmirGLO plasmids (Promega, Madison, WI, U.S.A.). Then, pmirGLO-DANCR-Mutant was generated by site-directed mutagenesis PCR with platinum pfx DNA polymerase as per the product manual, which acted as NC. Luciferase reporter plasmids and miR-216a-5p mimics/miR-NC mimics (miR-NC mimics) were cotransfected into DANCR-mutant cells (DANCR-MUT) and DANCR-wild type cells (DANCR-WT) by Lipofectamine 2000. After transfection 48 h, relative luciferase activity of cells was examined via a luminometer by Dual-Luciferase Reporter Assay System (Promega, Madison, WI, U.S.A.).

### Cell proliferation assay

The cell proliferation assay was performed using Cell Counting Kit-8 (CCK-8) method. MCF-7 and MDA-MB-231 cells were first transfected with siRNA-negative control (si-NC), si-DANCR, 216a-inhibitor, or si-DANCR+ 216a-inhibitor. Then the cells were seeded into 96-well plates and cultured in an incubator at 37°C with 5% CO_2_. After transfection 24, 48, 72, and 96 h, 10 μl of CCK-8 reagent (Dojindo, Japan) was added to each well and continued incubating for 2 h at 37°C. Finally, the absorbance values of all wells at 450 nm were measured by an enzyme immunoassay analyzer (Bio-Rad, Hercules, CA, U.S.A.).

### Cell migration and invasion assay

The migration and invasion of breast cancer cells were measured using wound-healing assay and transwell assay. Each kind of breast cancer cells was divided into four groups based on transfection: si-NC, si-DANCR, 216a-inhibitor, and si-DANCR+ 216a-inhibitor. For wound-healing assay, breast cancer cells were seeded in six-well plates and incubated until they grew to 90% confluence. Then, the media were removed and the monolayer cells were manually scraped using sterile pipette tip. At incubation 0 and 24 h, the width of the wounding scratches was observed and measured under a microscope (Leica, Germany). For transwell assay, cell-culture insert (8mm, BD Biosciences, U.S.A.) was first plated with Matrigel gelatin (BD Biosciences, U.S.A.) according to the manufacturer’s protocol. Then, breast cancer cells were resuspended in serum-free medium and planted in the top chamber. The normal medium containing 10% fetal bovine serums was added to the lower chamber. After incubation 24 h, the cells invaded into the lower chamber were fixed in 100% cold methanol and stained by 0.05% crystal violet. The number of invading cells in 5 randomly selected views was recorded.

### Western blot

Breast cancer cells were lysed using RIPA buffer (Sigma–Aldrich, St. Louis, U.S.A.) containing protease inhibitors cocktail (Roche, Diagnostics, Mannheim, Germany). The total protein was obtained by centrifuging with 12,000 ***g***, and the protein concentration was measured by BCA test. Then, equal amounts of protein was separated via SDS-PAGE and transferred to PVDF membrane (Millipore, Bedford, U.S.A.). PVDF membrane was blocked with 5% skim milk and then incubated with primary antibodies at 4°C. The primary antibodies were obtained from Abcam (Cambridge, MA, U.K.) and used with 1:1000 dilutions, including anti-E-cadherin, anti-Nanog, anti-SOX2, anti-OCT4, and anti-GADPH. Amongst these, GADPH acted as a loading control. Subsequently, the membrane was washed by PBS-T buffer (Sigma) and incubated with conjugated goat antirabbit IgG (Abcam) at room temperature for 2 h. Finally, the signals were measured using ECL detection kit (Beyotime Biotechnology, Shanghai, China).

### Xenograft animal study

First, MDA-MB-231 cells were transfected with lentiviral vectors (LV). shRNA plasmid directly targetting DANCR (sh-DANCR) and empty shRNA plasmid (sh-control) were constructed by Shanghai Genechem (China) and introduced into pFU-GW-RNAi vector that carried green fluorescent protein reporter gene driven by the U6 promoter. Then cancer cells were planted in six-well plates and infected with LV-sh-DANCR or LV-sh-control. Xenograft animal study was approved by the Institutional Animal Care and Use Committee of The Third Affiliated Hospital of Harbin Medical University and carried out based on the experimental protocols. Briefly, lentiviral-transfected cancer cells were injected subcutaneously into the left posterior flank of 6-week-old BALB/c mice (1 × 10^7^ cells per mouse) to obtain tumor growth model. At 8 weeks post injection, the mice were killed and the tumors were put out. The relative expression levels of DANCR and miR-216a in tumors were detected by qRT-PCR.

### Immunohistochemical staining

First, tumor tissue sections of mice were dried at 60°C for 1 h. Then, the sections were dewaxed in xylene and rehydrated in graded concentrations of alcohol. After that, the sections were treated by citrate buffer (pH 6.0) and autoclaved for 90 s at 121°C. After washing with PBS, sections were blocked with goat serum (Boster, Wuhan, China) for 30 min at room temperature and then incubated with Ki67 antibody (1:200, Bioss Antibodies, MA, U.S.A.) overnight at 4°C. Next, sections were washed by PBS and incubated using Polink-1 HRP DAB Detection System One-step polymer detection system (ZSGB-BIO, Beijing, China) for 20 min at room temperature. Finally, tissue sections were counterstained via hematoxylin.

### Statistical analysis

Each experiment was repeated at last three times, and all data were presented as the means ± S.D. Expression correlation assays were performed using Pearson’s coefficient correlation. The comparisons between groups were analyzed by two-tailed Student’s *t*-test or one-way ANOVA followed by the LSD *post hoc* test. If *P* value < 0.05, the result was considered to be statistically significant.

## Results

### DANCR was up-regulated in breast cancer patients

**Figure 1:**
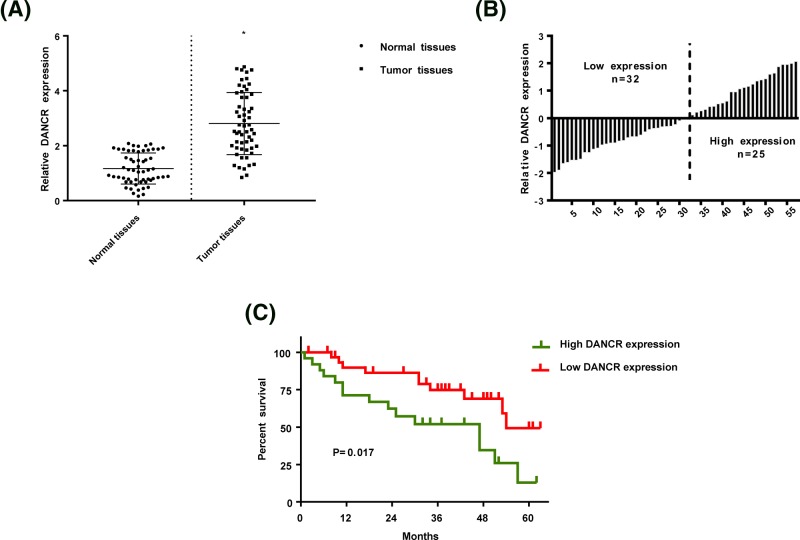
The expression of DANCR in TNBC patients (**A**) DANCR was up-regulated in tumor tissues compared with normal tissuses. (**B**) 57 TNBC patients were divided into low expression group and high expression group based on DANCR level. (**C**) The comparison of survival rate between low expression group and high expression group.

To explore whether DANCR disorder occurred in TNBC patients, qRT-PCR was performed to detect its expression level in breast cancer tissues and paired adjacent non-tumor tissues (n = 57). Figure 1A showed that DANCR was significantly up-regulated in tumor tissues compared with normal tissues, implying that DANCR might be related with breast cancer progress. Then the 57 TNBC patients were divided into two groups based on DANCR expression level: high expression group (n = 25) and low expression group (n = 32) (Figure 1B). We analyzed the overall survival of patients in the two groups and the results displayed that the percent survival in low expression group was obviously higher than that in high expression group (Figure 1C).

### Down-regulation DANCR could reduce cell proliferation and invasion

**Figure 2:**
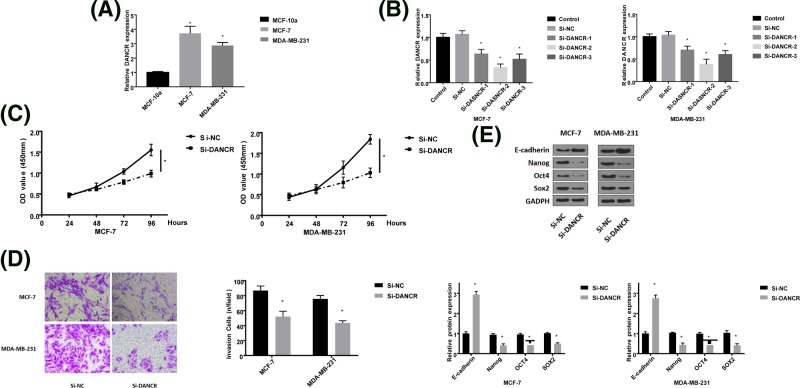
The influence of down-regulation DANCR on breast cancer cells (**A**) The expression level of DANCR in MCF-7, MDA-MB-231 cells. (**B**) Breast cancer cells were transfected with siRNA to down-regulate DANCR level. (**C**) The effect of DANCR knockdown on cell proliferation in breast cancer. (**D**) The influence of DANCR knockdown on cell invasion in breast cancer. (**E**) Relative expression levels of E-cadherin, Nanog, OCT4, and SOX2 after breast cancer cells were transfected with siRNA. (*P<0.05).

To study the influence of DANCR on breast cancer cells, cell transfection assay was performed. Before transfection, DANCR was also highly expressed in two breast cancer cells (MCF-7 and MDA-MB-231) (Figure 2A). After transfection, the inhibition effects of si-DANCR were detected by qRT-PCR. Figure 2B showed that si-DANCR-2 could inhibit DANCR expression better in the two cancer cells; thus, we chose it to use for subsequent experiments. Then CCK-8 and transwell assay were executed. As shown as Figure 2C,D, the decrease of DANCR could significantly suppress cell proliferation and weaken cell invasion. The data of western blot displayed that the knockdown of DANCR had abilities to increase E-cadherin expression and reduce the levels of Nanog, OCT4, and SOX2 (Figure 2E), which suggested that the effects of DANCR on cell proliferation and invasion might have close relationships with epithelial-mesenchymal transition (EMT).

### miRNA-216a-5p could interact with DANCR in breast cancer cells

**Figure 3:**
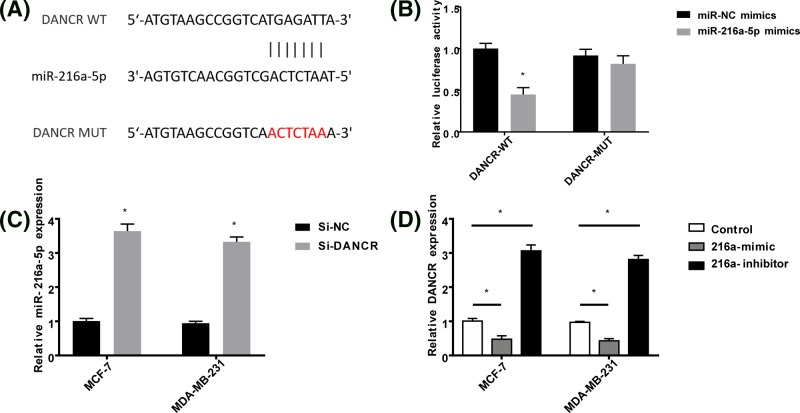
DANCR interacted with miR-216a-5p (**A**) A putative miR-216a-5p binding site was predicted by bioinformatics analysis. (**B**) The relative luciferase activity of DANCR-WT and DANCR-MUT co-transfected with miR-216a-5p mimics and miR-NC. (**C**) The expression level of miR-216a-5p in breast cancer cells transfected with siRNA-NC or siRNA-DANCR. (**D**) Relative DANCR expression level of breast cancer cells transfected with 216a-mimics or 216a-inhibitor. *P<0.05.

Bioinformatic analysis showed that in theory, DANCR had a binding site of miRNA-216a-5p (Figure 3A). To confirm this, luciferase reporter assay and qRT-PCR were performed. Figure 3B displayed that 216a-mimics could obviously reduce luciferase activity of DANCR-WT, while it had no distinct effects on DANCR-MUT. The results of qRT-PCR showed that breast cancer cells transfected with si-DANCR had higher level of miRNA-216a-5p, implying that inhibiting DANCR expression could lead to the increase of miRNA-216a-5p (Figure 3C). In addition, Figure 3D demonstrated that 216a-inhibitor had abilities to increase DANCR expression, while 216a-mimics had opposite effects on DANCR expression. The above data proved that miRNA-216a-5p could regulate DANCR level in breast cancer cells by combining with DANCR.

### DANCR regulated breast cancer cell’s proliferation, invasion, and migration by targetting miRNA-216a-5p

**Figure 4:**
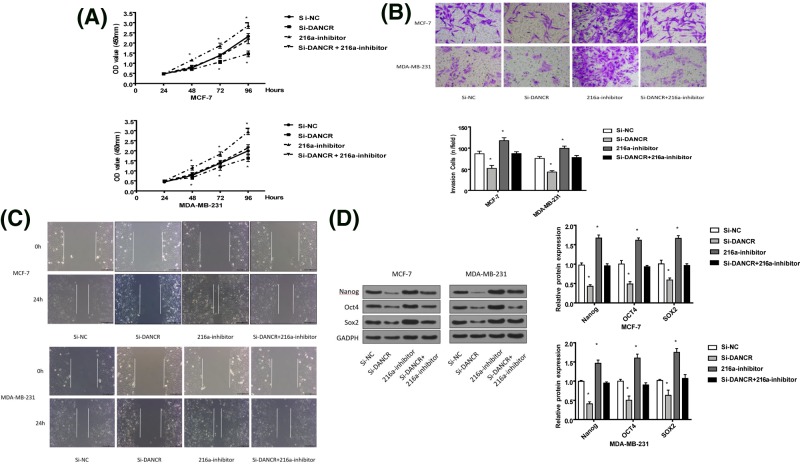
DANCR promoted cell proliferation, invasion, and migration by targetting miR-216a-5p in breast cancer (**A**) Low DANCR decreased cell proliferation, while 216a-inhibitor reversed it. (**B**) DANCR silencing reduced cell invasion, whereas 216a-inhibitor had reversal functions. (**C**) DANCR knockdown reduced cell migration, while 216a-inhibitor had reversal functions. (**D**) DANCR knockdown significantly decreased the level of Nanog, OCT4, and SOX2, while 216a-inhibitor had reversal functions. *P<0.05.

To further research whether the effects of DANCR on breast cancer cells were related with miRNA-216a-5p, MCF-7, and MDA-MB-231 cells were transfected with si-NC, si-DANCR, 216a-inhibitor, or si-DANCR+ 216a-inhibitor. The result of CCK-8 exhibited that the cells, co-transfected with si-DANCR+ 216a-inhibitor, had higher optical density values than those transfected by only si-DANCR, which implied that 216a-inhibitor could inhibit the effect of si-DANCR on cell proliferation (Figure 4A). In addition, transwell assay and wound-healing assay indicated that the suppressive effects of si-DANCR on cell invasion and migration also could be weakened by 216a-inhibitor to some extent (Figure 4B,C). Moreover, the result of western blot further indicated that 216a-inhibitor could increase the levels of Nanog, OCT4, and SOX2, as well as reduce the functions of si-DANCR on the three proteins (Figure 4D). The above analysis demonstrated that the 216a-inhibitor reversed the outcomes caused by DANCR knockdown in certain degree, which indicated that DANCR might promote cell proliferation, invasion, and migration by targetting miR-216a-5p.

### Reducing DANCR level could inhibit tumor growth

**Figure 5:**
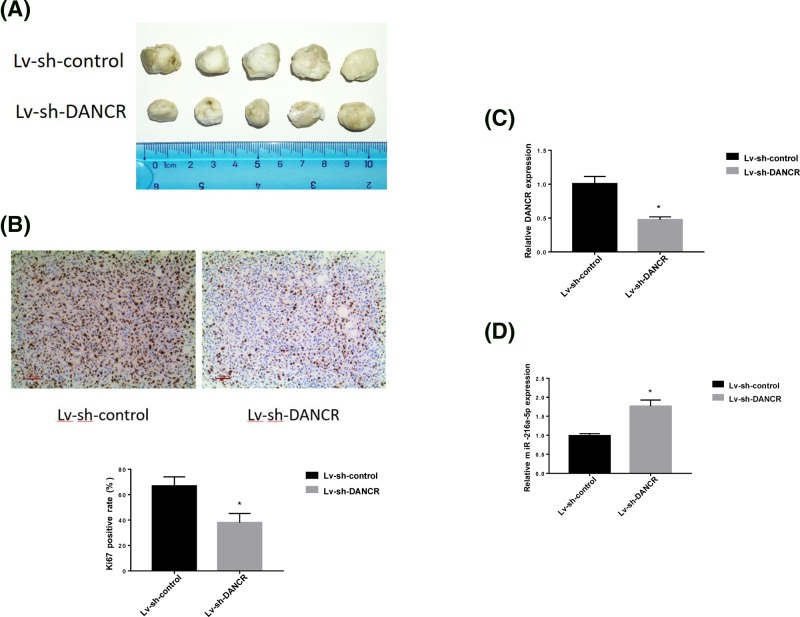
Down-regulation DANCR inhibited tumor progression *in vivo* (**A**) The tumor sizes after MCF-7 cells were inoculated for 8 weeks. (**B**) The Ki67 activity in tumors. (**C**) DANCR expression level in tumor. (**D**) Relative miR-216a-5p level in tumor. *P<0.05.

Xenograft animal study was performed to validate whether DANCR knockdown could inhibit breast cancer cell growth and migration *in vivo*. LV-sh-DANCR-transfected MDA-MB-231 cells were subcutaneously injected into nude mice for 8 weeks. As shown as Figure 5A, the tumor sizes of LV-sh-DANCR group were smaller than those of control. Immunohistochemical staining revealed that Ki67 level was obviously lower in cells transfected with LV-sh-DANCR than those transfected with LV-sh-control (Figure 5B), which suggested that down-regulation DANCR could also reduce cancer cell proliferation and migration *in vivo*. Moreover, the results of qRT-PCR also displayed that LV-sh-DANCR was able to decrease DANCR level and increase miRNA-216a-5p level in tumors (Figure 5C,D), proving that DANCR expression level had close relationships with tumor growth and migration.

## Discussion

Breast cancer is the second leading reason of cancer-caused death amongst women worldwide. With the advance of early cancer detection by ultrasonography and mammography and the development of multiple targetted therapeutic drugs for hormone and growth receptors, the 5-year survival rate has been nearly 90% [21]. Nevertheless, the treatment for TNBC still faced great challenge due to its highly aggressive character and lack of available target markers for systemic therapy [22]. Therefore, more works are necessary to deeply understand the molecular mechanism of this type breast cancer, as well as identify new biomarkers to develop better therapeutic approach. Currently, plenty of studies have found that lncRNAs play important roles on various cellular processes and had close relationships with many diseases, including variety of human cancers [10]. DANCR, one newly reported lncRNA, has been found that it was very necessary for the de-differentiation of epidermal cells and played important regulatory roles in osteoblast differentiation [19, 23–25]. In addition, recent researches have also proved that DANCR exerts oncogenic functions in many cancers, for example, it could promote cell proliferation and invasion by miR-758-3p [19]. In the present study paper, we found that DANCR was obviously overexpressed in TNBC tissues and breast cancer cells compares with relative normal tissues and breast cells, and higher DANCR level suggested poorer prognosis.

To explore DANCR functions on TNBC, we selected MDA-MB-231 cells used for further experiments. The results of CCK-8 and transwell assay showed that down-regulation DANCR could obviously weaken cell proliferation and invasion, while its overexpression had opposite influences. In addition, animal experiment also demonstrated that high level of DANCR had promoting roles on breast tumor growth and Ki67 activity. Our results were consistent with previous studies, suggesting that DANCR was potential to be a new biomarker used for TNBC diagnosis and prognosis. It is well known that cancer cells have abilities to leave their epithelial state and acquire features of mesenchymal cells, which leads to tumor growth and metastasis. In this process, EMT was orchestrated through various mechanisms [22]. E-cadherin, Nanog, OCT4, and SOX2 were important factors for EMT, and previous paper has been reported that the four proteins were related with TNBC progress [26]. Western blot displayed that DANCR knockdown could significantly increase E-cadherin and decrease Nanog, OCT4, and SOX2 in MDA-MB-231 cells, implying that DANCR had influences on cell proliferation and invasion by regulating EMT.

However, its molecular mechanism was unknown in breast cancer. Recently, several studies brought forward a competing endogenous RNA (ceRNA) hypothesis that lncRNAs could communicate with other protein-coding RNA transcripts to modulate the level or functions of miRNAs via shared miRNA binding sites [27, 28]. Surprisingly, latest researches reported that DANCR worked as a ceRNA in some cancers. For examples, DANCR exerted tumor promoter in non-small cell lung cancer by directly targetting miR-758-3p; and DANCR controlled mTOR expression to modulate tumor progression via sponging miR-496 [19, 29]. To explore whether DANCR acted as a ceRNA in breast cancer, miR-216a-5p was identified by bioinformatic analysis. The results of luciferase activity assay and qRT-PCR exhibited that miR-216a-5p was a regulation target of DANCR and their expression levels could adjust to each other. In addition, 216a-inhibitor could improve the levels of Nanog, OCT4 and SOX2, and had inhibiting effects on si-DANCR in cell proliferation, invasion, and migration for MDA-MB-231 cells. Moreover, *in vitro* research suggested that miR-216a-5p was up-regulated upon DANCR down-regulation. The above analysis demonstrated that DANCR exerted oncogenic functions by targetting miRNA-216a-5p in breast cancer.

In order to better research the functions and mechanism of DANCR on breast cancer, one ER-positive breast cancer cell line–MCF-7 was also selected to verify whether it also had promoting functions on other subtype of breast cancer. Regrettably, the results displayed that DANCR played same effects on MCF-7 cells by targetting miR-216a-5p, which suggested that it probably played same roles on some subtypes of breast cancer, including TNBC and ER-positive breast cancer. Surely, there were some limitations in our study. We did not select another TNBC cell line to verify our results, so the results might be not extrapolated to all TNBC.

In conclusion, our work found that DANCR had promoting functions on cell proliferation, invasion, and migration in breast cancer through working as ceRNA to target miR-216a-5p, which indicated that the new axis of DANCR/miR-216a-5p might provide a potential therapy target for breast cancer treatments, not only TNBC but also ER-positive breast cancer. Moreover, these results also indicated DANCR was promising to be a novel biomarker used for breast cancer diagnosis and prognosis.

## Availability of data and materials

The analyzed datasets generated during the study are available from the corresponding author on reasonable request.

## Abbreviations

CCK-8: cell counting kit-8
cDNA: complementary DNA
ceRNA: competing endogenous RNA
DANCR: differentiation antagonizing non-protein coding RNA
EMT: epithelial-mesenchymal transition
HER-2: human epidermal growth factor receptor 2
LV: lentiviral vector
NC: Negative control
PR: progesterone receptor
si-NC: siRNA-negative control
TNBC: triple-negative breast cancer
